# Triclosan: Current Status, Occurrence, Environmental Risks and Bioaccumulation Potential

**DOI:** 10.3390/ijerph120505657

**Published:** 2015-05-22

**Authors:** Gurpreet Singh Dhillon, Surinder Kaur, Rama Pulicharla, Satinder Kaur Brar, Maximiliano Cledón, Mausam Verma, Rao Y. Surampalli

**Affiliations:** 1 INRS-ETE, Université du Québec, 490, Rue de la Couronne, Québec, QC G1K 9A9, Canada; E-Mails: garrydhillons9@gmail.com (G.S.D.); surinder_dhillons@yahoo.ca (S.K.); Pulicharla.Rama@ete.inrs.ca (R.P.); Maximiliano.Cledon@ete.inrs.ca (M.C.); 2 Department of Mycology & Plant Pathology, Institute of Agricultural Sciences, Banaras Hindu University (BHU), Varanasi-221005, India; 3 CONICET-IIMyC, National Council of Scientific and Technical Research, C1033AAJ Buenos Aires, Argentina; 4 CO_2_ Solutions Inc., 2300, Rue Jean-Perrin, Québec, QC G2C 1T9, Canada; E-Mail: mausamverma@yahoo.com; 5 Department of Civil Engineering, University of Nebraska-Lincoln, N104 SEC P.O. Box 886105, Lincoln, NE 68588, USA; E-Mail: surampalli.rao@giees.org

**Keywords:** degradation by-products, dioxins, emerging contaminants, personal care products, triclosan, toxicity

## Abstract

Triclosan (TCS) is a multi-purpose antimicrobial agent used as a common ingredient in everyday household personal care and consumer products. The expanded use of TCS provides a number of pathways for the compound to enter the environment and it has been detected in sewage treatment plant effluents; surface; ground and drinking water. The physico-chemical properties indicate the bioaccumulation and persistence potential of TCS in the environment. Hence, there is an increasing concern about the presence of TCS in the environment and its potential negative effects on human and animal health. Nevertheless, scarce monitoring data could be one reason for not prioritizing TCS as emerging contaminant. Conventional water and wastewater treatment processes are unable to completely remove the TCS and even form toxic intermediates. Considering the worldwide application of personal care products containing TCS and inefficient removal and its toxic effects on aquatic organisms, the compound should be considered on the priority list of emerging contaminants and its utilization in all products should be regulated.

## 1. Introduction

Triclosan (TCS, 5-chloro-2-(2,4-dichlorophenoxy) phenol) is a synthetic, broad-spectrum antimicrobial agent. It has antibiotic and antimycotic properties [1]. Triclosan also blocks fatty acid synthesis by inhibiting enoyl reductase enzyme. TCS is categorized as a halogenated aromatic hydrocarbon having phenolic, diphenyl ether and polychlorinated biphenyl (PCB) substructures [2]. Its chemical structure is a halogenated biphenyl ether which confers it chemical properties related to many toxic compounds such as PCBs, polybrominated diphenyl ethers, bispenol A and dioxins [3].

The worldwide annual production of TCS in 1998 was approximately 1500 tonnes, out of which about 350 tonnes and more than 450 tonnes were utilized in Europe and USA, respectively [4,5]. The main release of TCS into the environment is due to personal care products containing around 0.1% to 0.3% (w/w) TCS [6,7]. Such products are externally applied to the human body, thus TCS is generally not subjected to metabolic alteration. Moreover, it is usually released into the domestic wastewater, thus ending up in local wastewater treatment plants (WWTP). Poor solubility and high adsorption of TCS to solids results in its removal from WWTP effluent up to 99%. [8,9]. The high log K_ow_ value of 4.76 for TCS suggests high sorption potential and it adsorbs onto the settled sewage sludge [10,11] which may be amended to agricultural soils [12,13]. Thus, the most important sources of TCS in the environment are use of biosolids as agro-fertilizers [14]. The chemical properties of TCS suggest its possible bioaccumulation and further environmental persistence (Table 1).

Currently, TCS and its degraded byproducts are found throughout the environment, including soil, surface waters, and human breast milk [14,15,16,17,18]. The continuous detection of TCS and its degradation products has led to debate on safety, effectiveness and regulation of TCS usage. Various studies shed light on the emerging health concerns related to the use of TCS, such as microbial resistance, dermal irritations, endocrine disruption, higher incidence of allergies, altered thyroid hormone metabolism and tumors development due to TCS and its by-products [19,20,21]. Unlike other emerging contaminants (ECs), such as organochlorine compounds, pharmaceutically active compounds (PhACs) and endocrine disrupting compounds (EDCs), TCS is not considered as a chemical pollutant with high priority concerns. Low acute toxicity and assumption of not to show chronic side effects, TCS usage is not well regulated [22,23]. This leads to widespread use of TCS in various household products, thus causing an increase in TCS concentration in the aquatic and terrestrial environment.

**Table 1:** General properties of TCS.

CAS No.	3380-34-5
Structure	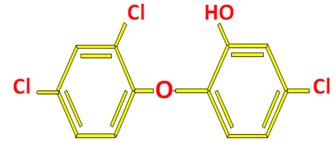
Molecular formula	C_12_H_7_Cl_3_O_2_
Trade name	Irgasan DP 300, FAT 80′023, CH 3565, GP41-353, Irgacare MP (the pharmaceutical grade of TCS, >99% pure) and Ster-Zac
General classification	Non-prescription compound
Possible use	Antimicrobial, antiseptic and disinfectant
Nature	Hydrophobic
Molecular weight	289.54
Dissociation constant (pKa) (20 °C)	8.14
Henry constant (H_c_) (atm mol^−1^·m^−3^)	1.5 × 10^−7^ (25 °C)
Octanol-water Partition coefficient (log K_ow)_	4.76
Sorption coefficient (K_oc_)	18408
Solubility	12 mg·L^−1^ (25 °C)
Vapor pressure	5.2 × 10^−6^ Pa (mm Hg at 20 °C)
Bioconcentration factor (BCF)-	2.7–90 (aquatic organisms)
Photodegradation (half-life in aqueous solution)	41 min
Biodegradation (half-life in aerobic soil)	18 days
Biodegradation (anaerobic condition)	No degradation within 70 days
Degradation products of TCS	Methyl TCS, dioxins, chlorophenols, chloroform

Similar antimicrobial activity of TCS to antibiotics and its toxicity data demand regular monitoring of its concentration in the environment, along with its safe and regulated use in the consumer products. This article provides a comprehensive literature review on TCS, its occurrence in wastewaters, biosolids, aquatic and terrestrial environment, its removal potential, toxicity levels in humans, wildlife and other aquatic organisms, its bioaccumulation potential and intermediate products. The review also addresses the research gaps in concerns related to long term exposure to TCS.

## 2. Physico-Chemical Properties of TCS Affecting Removal

The removal of organic substances, such as TCS after release into environment depends on various physico-chemical properties of the compound. For instance, the sorption of organic compounds on sludge during wastewater treatment processes plays an important role. Depending on their log K_ow_ values, the hydrophobic substances may adsorb onto settled sludge during primary sedimentation step in WWTP. The different physico-chemical characteristics of TCS governing its removal efficiency in conventional activated sludge treatment plants are given in Table 2. As evident from Table 2, the adsorption potential of TCS is high due to a high log K_ow_. The high K_ow_ value of TCS is also indicator of its bioaccumulation potential. Another important property governing the removal of organic substances is their volatility. Triclosan is also non-volatile (5.3 × 10^−4^ Pa at 20 °C) and is moderately soluble in water (10 mg·L^−1^ at 20 °C). Moreover, it does not hydrolyze easily [24]. Normally, the substances with a Henry’s constant (H_c_) ≥ 10^−3^ atm·mol^−1^·m^−3^ will easily be removed by volatilization. Hence, the volatilization losses of specific substances during wastewater treatment can be predicted based on Henry’s constant value and Hc/Log K_ow_ ratio [11].

**Table 2:** Removal potential of TCS during wastewater treatment process depending on different physico-chemical properties.

Physico-Chemical Property	Removal Potential of TCS
Adsorption potential	
Log K_ow_ ≤ 2.5	Low sorption potential
2.5 < Log K_ow_ < 4	Medium sorption potential
Log K_ow_ ≤ 4	High sorption potential^TCS^
Volatilization potential	
H_c_ > 1 × 10^4^ and H_c_/Log K_ow_ >1 × 10^9^	High volatization potential
H_c_ < 1 × 10^4^ and H_c_/Log K_ow_ <1 × 10^9^	Low volatization potential^TCS^

The removal potential of TCS is given with a superscript; H_c_/Log K_ow_ ratio of TCS is 8.67 × 10^14^.

**Figure 1:**
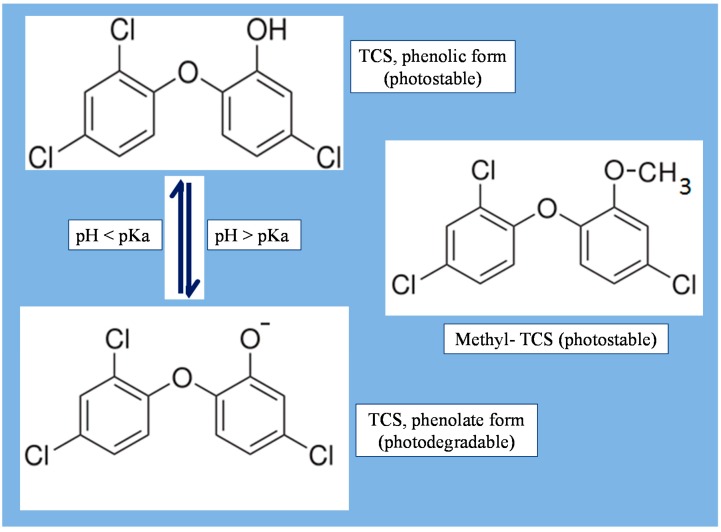
Molecular structures of TCS and its environmental transformation product, methyl-TCS.

TCS is a chlorinated phenoxyphenol with a pKa of 8.1 and is photodegradable into its photostable phenolate form (Figure 1). The phenolate-triclosan predominates when the natural water pH > 8.1 and it converts into its neutral phenolic form if the water pH is below 7.9. In addition to pH, co-occurrence of dissolved compounds such as metals and organic matter may possibly affect photosensitivity of TCS [24]. Hence, the complex matrix of wastewater affects the efficiency of photodegradation of TCS in WWTP [25].

## 3. Current Scenario of TCS Use and Safety

**Figure 2:**
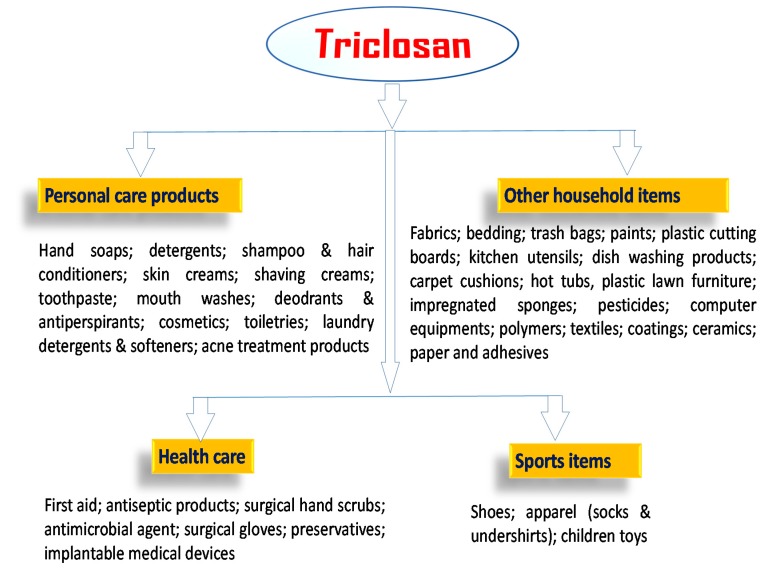
Various applications of triclosan.

Generally, TCS comes in the form of white powder. TCS has a weak aromatic, phenolic scent as it is a chlorinated aromatic compound. Ever since its invention, TCS has been widely used in numerous consumer products as illustrated in Figure 2 [6,8,10,12,26]. It is used as an active ingredient in dental products since 1980s in Europe and the mid-1990s in the United States after approval by the Food and Drug Administration [27]. More specifically, TCS is used in numerous personal care products, such as toothpastes, antibacterial soaps (bars and liquids), dishwashing liquids, deodorant soaps (bars and liquids), cosmetic and antiseptic products, and antiperspirants/deodorants [28]. Triclosan is also used in other consumer products, such as kitchen utensils, toys, bedding, clothes, fabrics, and trash bags.

The concentration of TCS recommended by various government agencies to be used in various consumer products is given in Table 3. In 1989, the European Community Cosmetic Directive approved TCS usage as a preservative in cosmetics and toiletries up to 0.3% [28]. According to FDA, up to 0.3% TCS is permitted in toothpaste [29]. Similarly, as per the National Library of Medicine’s Household Product Database, TCS concentrations were reported to range from 0.1% to 0.3% in liquid hand soaps [30].

**Table 3:** Recommended levels of TCS in various consumer products (Adapted from [25].

Type of TCS-Based Product	TCS Concentration (%)	Reference
**Oral care products**		
Toothpaste	0.3	[29]
Mouth wash solutions	0.03	[31]
**Dermally applied products (rinse off)**		
Skin cleansers	0.3	[28]
Liquid hand soap	0.1–0.45	[32]
Dishwashing detergent	0.1	[30]
**Dermally applied products (leave on)**		
Body lotion	0.3	[28]
Facial Moisturizer	0.3	[28]
Deodorant/antiperspirants	0.3	[28]

According to the FDA monograph for health care antiseptic drug products, which covered antibacterial soap products containing TCS, the recommended limits are up to 1% TCS for use in antiseptic washes and surgical hand scrubs in health care settings [33]. According to Governmental regulations in the European Union (EU) and the United States, only specified amount of triclosan can be used in some cosmetic and PCPs.

TCS possesses a broad range of antimicrobial activity that encompasses several, types of non-sporulating bacteria and a few fungi, such as *Plasmodium falciparum* and *Toxoplasma gondii* [19,34]. At low concentrations, TCS inhibits the growth of microorganisms; at higher concentrations, it kills microorganisms. Different microorganisms show varied response to TCS as provided in Table 4. Triclosan blocks the active site of enoyl-acyl carrier protein reductase enzyme (ENR) thus impairing the production of bacterial lipids [35]. In consequence, cell membranes are not properly produced and bacterial proliferation stops. Therefore, only a small TCS dose is required to inhibit bacterial growth. As humans lack ENR enzyme, TCS has been considered harmless to them.

Studies carried out by FDA found that TCS-fluoride paste prevented tooth deformities, such as gingivitis, tartar and plaque in a way that was superior to fluoride-only toothpastes. Over the last 30 years, TCS has also been successfully used as an antimicrobial agent in hospitals and for other biomedical purposes. The successful control of methicillin-resistant *Staphylococcus aureus* (MRSA) outbreaks in several clinical settings using TCS based products [36,37]. This led to the recommendation of showering/bathing with 2% TCS for the decolonization of patients whose skin is carrying MRSA [38]. However, susceptibility of MRSA strains to TCS has changed little over the last decade [39]. Later on there has been no relation found between TCS response in MRSA and other strains of *S. aureus* and antibiotic susceptibility or resistance [40].

**Table 4:** Different microorganisms affected by the antimicrobial action of TCS.

Target Microorganisms	Effective Concentrations	Reference
**Most sensitive strains**		
Staphylococci, some Streptococci, some mycobacteria, *Escherichia coli*, *Klebsiella pneumonia*, *Klebsiella* spp., *Enterobacter* spp., *Acinetobacter* spp., *Proteus* spp. and *Proteus mirabilis*, *Plasmodium falciparum*, *Toxoplasma gondii*	0.01 mg·L^−1^ to 0.1 mg·L^−1^	[33] [19]
**Less sensitive strains**		
Methicillin-resistant *Staphylococcus aureus* (MRSA) strains	0.1–2 mg·L^−1^	[40,142]
Enterococci	-	[49]
**Highly resistant strains**		
*Pseudomonas aeruginosa*, *Clostridium difficile*	-	[49,143]

The American Medical Association (AMA) has raised concerns about the use of TCS and some other antimicrobial agents in consumer products [41]. The AMA has encouraged the FDA to study the issue on the safety and effectiveness of antimicrobials including TCS. The progress of the current FDA evaluation will be monitored by the AMA on regular basis. The AMA also indicated that further research is required on the introduction of antimicrobials in massive consumer products. In 2009, the American Public Health Association (APHA) proposed that it would recommend the banning of TCS for household and non-medical uses. However, no further action has been taken as yet. Regardless of current efforts to review and regulate the proper use of TCS, a scientific debate lingers on its potential adverse impact on human health, environment and potential association to microbial resistance.

## 4. Emergence of Microbial Resistance to TCS

The overuse of anti-microbial products may lead to increased resistance among bacteria. Considering the published studies, there is a dilemma whether TCS does or does not encourage the development of antibiotic resistance. Triclosan-resistant bacteria can be produced readily by their *in vitro* exposure to increasing TCS quantities and the consequent development of resistant colonies [42].

The mechanism of microbial resistance to TCS has been described by various researchers [43,44]. According to the authors, the resistance can be attributed to: (1) overproduction of targets/amplification or; (2) modification of target. Gomez-Escalda *et al.* [45] found that a combination of membrane impermeability and efflux were responsible for the increased insusceptibility of *E. coli* isolates to TCS. Various studies demonstrated the development of microbial resistance following exposure to TCS [44,46,47]. Reiss *et al.* [48] described the induction of expression of an efflux pump in *P. aeruginosa* following TCS exposure, resulting in high-level resistance to TCS and the antibiotic, ciprofloxacin. In *E. coli*, resistance can be attributed to either overexpression of the TCS target enzyme enoyl reductase or to changes in cellular permeability [49]. The most resistant bacteria have slow growth rate as compared to sensitive bacteria. On the contrary, *E. coli* resistant to TCS actually possess enhanced growth rates. The intrinsic resistance of *P. aeruginosa* to TCS can be attributed to: (1) a non-susceptible enoyl reductase; (2) an outer membrane permeability barrier or; (3) pumping of the drug from the cell interior to its exterior [50]. The latter has been stated as the major reason for TCS insusceptibility [51,52] in *P. aeruginosa*. MRSA strains, meanwhile, may or may not show decreased sensitivity to triclosan [50,53]. Study conducted by Fan *et al.* [54] demonstrated that all *S. aureus* strains with decreased sensitivity overproduced the enzyme *Fab I* by 3–5 fold and moreover, mutations in *Fab I* were found in the most resistant strains.

Major concern is that the mode of action of TCS and its target site in the microbes is similar to antibiotics. The enzymes enoyl reductase (product of *Fab I* among Gram-positive and Gram-negative bacteria and *Inh A* in Mycobacterium e.g., *M. smegmatic and M*. *tuberculosis*) involved in fatty acid biosynthesis are the targets for a number of structurally unrelated drugs, including TCS. For instance, isoniazid an antibiotic used to treat tuberculosis that targets the same enzyme system [55]. Thus, TCs belongs to the group of drugs, such as isoniazid (tuberculosis) and diazoborine (experimental antibiotic) which target the enzyme enoyl reductase. Hence, a mutation in the enzyme may lead to resistance to TCS and these drugs. The overuse of TCS may result in the development of cross-resistance to antibiotics, and thereby the emergence of bacterial strains resistant to both TCS and antibiotics [56].

The laboratory studies play an important role in evaluating mechanisms of action and resistance to biocides, including TCS. These studies are mostly related to a wide range of medical applications [49,57]. Various researchers have purported to demonstrate a correlation between the use of biocides including TCS and antibiotic resistance [55,58,59]. On the contrary, few authors advocated that TCS use should be regulated as all other biocides [8,60]. There was no relationship found between TCS application and antibiotic tolerance in methicillin-resistant *Staphylococcus aureus* and *P. aeruginosa* during a 10 year study conducted by [32]. Marshall *et al.* [61] reported no differences in overall titers of bacteria or frequencies of antibiotic resistance in a snap-shot investigation among homes using or not using bactericide products. Similarly, a comprehensive study by Cole *et al.* [62] found no relationship between the use of biocides including TCS and antibiotic resistance in homes with use/no use of biocidal agents.

There was a concern that the use of TCS in dental hygiene products results in the development of TCS-resistant bacteria that are less sensible to common antibiotics. In view of this, an expert panel review concluded that there was no evidence of resistance development in the opportunistic or pathogenic microorganisms following the exposure to TCS [63]. The interim use of TCS containing dental hygienic products does not affect the stable microflora of the mouth or changes the susceptibility of Streptococci to antibiotics. However, chronic exposure to TCS demonstrated less significant decrease in antibiotic susceptibility in dental bacteria [64]. Usually, the introduction of bacteriostatic compounds to hinder plaque growth is seen as necessary [65]. Although TCS resistance in laboratory experiments may be linked with changes in antibiotic susceptibility, but comprehensive environmental investigations have not yet clearly established any relationship between TCS usage and antibiotic resistance. It is now well known that laboratory findings do not always apply in the real world environment [42].

In general, bacterial resistance to disinfectants is not a new phenomenon. The phenomenon of decreased susceptibility to various disinfectants was being described over a century ago by various researchers as thoroughly reviewed by Russell, [66], before the introduction of TCS. The study conducted by Tan *et al.* [67] indicated that resistance to TCS and other biocides is increasing. This conclusion was generally based upon minimum inhibitory concentrations (MIC) in laboratory experiments rather than bactericidal estimations. There might not be a correlation between a poor rate of kill and sensitivity at MIC level [49]. The use of MIC investigation to study emerging bacterial resistance is important as it can indicate a trend towards some resistance properties [40,68]. As resistance develops in a step-wise manner, it is judicious to conserve use and continued surveillance of susceptibility to antimicrobials.

## 5. Toxicity of TCS

Triclosan possesses broad-spectrum antimicrobial action and has been classified as a Class III drug (compounds with high solubility and low permeability) by FDA [69]. Due to environmental concerns, TCS was declared as Priority Existing Chemical for full assessment under the *Industrial Chemicals (Notification and Assessment) Act, 1989* (the Act) in the *Chemical Gazette* of 6 May 2003 [70]. Some signs of it have been already reported as TCS was not only found in WWTPs, but even in urine, plasma and breast milk in humans [20,71,72]. Studies have thus yielded contradictory findings regarding links between TCS and adverse health impacts in humans and animals.

### 5.1. Toxicity in Humans

Absorption, distribution, metabolism and excretion are rapid in the case of TCS in human body. TCS is metabolized to glucuronide and sulfate conjugates (phase II metabolism) and are primarily excreted via urine. These hydrophilic conjugates of TCS limit the bioaccumulation of TCS. Some studies indicated that TCS is comparatively non-toxic to humans and other mammals. Conversely, studies indicated that TCS exposure resulted in contact dermatitis, or skin irritation [73]. A photo-allergic dermatitis (PACD) reaction can be triggered when the skin comes in contact with TCS and is further exposed to sunlight [74]. PACD can result in symptoms, such as eczematous rash on the body parts with combined TCS and sunlight exposure. According to the claims made by various manufacturers of TCS-containing toothpaste and soaps, the active ingredient continues to work even up to 12 h after use. This prolonged exposure to TCS in turn increases the risk of PACD.

Triclosan has been found in urine, plasma, and breast milk of humans [16,20,75,76], but typically without attribution to specific sources of TCS exposure. High levels of TCS were found in 60% of human milk samples indicating the absorption potential of TCS into the body [15]. According to National Health and Nutrition Examination Survey (NHANES) data collected during 2003–2004, TCS was found in 75% of the analyzed urine samples [76,77]. The urinary data were collected for adult men and women and children between the ages of 6 and 11. NHANES is an ongoing annual survey conducted since 1999 by the US Centers for Disease Control and Prevention (CDC) aimed to collect data on selected chemicals, including TCS. This data is used to evaluate the nutrition quality and general health of the US population. Moreover, due to lipophilic nature of TCS, it may bioaccumulate in fatty tissues. Nevertheless, no study until date has established the carcinogenic, mutagenic, or teratogenic effects of TCS.

Another area of concern is related to the hypothesis that TCS augments the production of chloroform. A study carried out by Fiss *et al.* [78] described that TCS may involve in the generation of chloroform, under certain conditions can almost double the chloroform formation in the drinking water treated with chlorine. On the contrary, studies [79] showed that there was no production of measurable quantities of chloroform within a normal tooth-brush when using toothpaste containing TCS and normal chlorinated drinking water. According to US EPA classification, chloroform is a possible human carcinogen. As a consequence, there was a campaign in UK underlining the potential of TCS to cause cancer, although Hao *et al.* studies [79] revealed that the amount of chloroform generated was lower in volume. Meanwhile, TCS in household dishwashing soaps reacts with chlorinated H_2_O to produce significant quantities of chloroform, a probable human carcinogen [80].

### 5.2. Toxicity in Animals and Other Organisms

The toxic effects of TCS were also studied in various animal models. For instance, its negative effect on the metabolism of thyroidal hormones causes hypothermia and an overall depression of the central nervous system (CNS) of mice [81]. The exposure to 0.03 mg·L^−1^ TCS was associated with induction of the expression of the metamorphic genes in tadpoles, which induced their premature metamorphosis [82]. Similarly, the study carried out by Kumar *et al.* [83] interrelated TCS exposure with decreased sperm production in male rats. The authors proposed the hypothesis that TCS blocks the metabolism of thyroid hormone as it presents a structure similar to the thyroidal hormone in regards to the binding of the specific receptors. Later, the endogenous hormones cannot bind to the occupied receptors.

Its close structure resemblance to certain estrogens triggered masculinization of secondary characters in rice fishes [84]. A recent study by James *et al.* [85] pointed out that TCS can inhibit the estrogen sulfotransferase activity in sheep placenta which would cause negative effects in the fetus development. Although toxicity reports in humans from chronic usage of PCPs containing TCS as an active ingredient are not available, still it has been widely studied in laboratory animals. During chronic oncogenicity studies in mice, rats, and hamsters, treatment-related tumors were found only in the liver of male and female mice [23]. Application of the human relevance framework advocated that these tumors arose due to a mode of action which is not considered to be pertinent to humans [23]. However, Yueh *et al.* [86] found that long term exposure to TCS in mice enhances hepatocellular carcinoma. This mechanism of TCS induced liver carcinoma in mice and it should be evaluated as these findings strongly support the relevance of TCS toxicity to humans.

Studies have also demonstrated that TCS accumulates in mice tissue with bioaccumulation factors of 3700–8400 [87]. This data indicates that fish contains concentrations thousands of fold higher than those found in the water column. Moreover, the bacterial transformation product of TCS in wastewater, methyl TCS is relatively lipophilic and stable in the environment, making it more likely to bioaccumulate in fatty tissue and will not photodegrade [88]. The lipid-based concentrations of methyl TCS detected in fish were considerably higher than the concentrations in lake water, indicating significant bioaccumulation of the compound. For aquatic organisms, the potential uptake mechanisms of lipophilic contaminants are direct uptake from water through exposed surfaces, mainly gills (bioconcentration), and also through the consumption of food (biomagnification) [21]. James *et al.* [89] demonstrated that demethylation of methyl TCS was slower than TCS conjugation in cattle fish. The bioaccumulation and slow conversion of methyl TCS in lower level consumers could serve as potential carriers of triclosan from the environment to higher level consumers in food chain.

The structure and the function of algal communities in ecosystems receiving treated wastewater effluent may be affected by TCS contaminated wastewaters [90]. These alterations may result in shifts in nutrient processing capacity and natural food web structure of these streams. TCS was also identified as the responsible key pollutant for the observed effects on growth of the green algae, *Scenedesmus valuolatus* under realistic exposure conditions [91]. Various studies investigated the toxicity of TCS on higher aquatic organisms [92,93,94,95]. Acute toxicity values ranged from 1.4 to 3000 μg·L^−1^ with EC_50_ values for crustaceans (*Daphnia magna* mortality at 390 μg·L^−1^), insects (*Chironomus tentans* survival at 3000 μg·L^−1^), fish (*Pimephales promelas* mortality at 260 μg·L^−1^), higher plants (*Lemna gibba* growth inhibition at 62.5 μg·L^−1^) and microalgal species (*Scenedesmus subspicatus* growth inhibition at 1.4 μg·L^−^^1^, *Skeletonema* sp. at 66 μg·L^−1^). Moreover, the standard test organism, *Selenastrum capricornutum* (growth inhibition at 4.7 μg·L^−1^) was reported to be 30-fold more sensitive to TCS than the bacterium *Vibrio fischeri* (bioluminescence inhibition at 150 μg·L^−1^) [96]. The microalgae were found to be the most sensitive organism to TCS [92,94,97]. With the increasing concentrations of TCS in the environment, bacterial strains are more likely to adapt by developing resistance [59]. TCS has various important medical applications, thus the future goal must be to retain these important applications while eliminating the unnecessary ones for its safe use.

All toxicity studies on TCS highlight the risks and suggest ban on TCS usage. In consequence, the FDA proposed, for comprehensive assessment of TCS toxicity on human health and animals, to regulate its further usage in consumer products until more information is available. Even though this proposal does not include environmental fate of TCS, this factor should be included in complete profiling of any chemical introduced into consumer products. In this sense, in 2010, more than 80 organizations petitioned EPA to ban TCS usage beyond pesticides. Minnesota has banned sale of any cleaning product (soaps) that contains triclosan on 16 May 2014. This ban makes the most manufacturers to phase out triclosan until early 2017. In 2013, FDA announced that final action on TCS usage in soaps will be taken by 2016 across the world. To complete the North American scenario, in Canada, approximately 1730 products including personal care products, cosmetics and health products containing triclosan were reported in 2011. Some reports indicate that triclosan would be a wide ranging contaminant in Canada. Therefore, from 2015 on, Health Canada is in the process to ban TCS.

## 6. Occurrence of TCS in Aquatic and Terrestrial Environment

Incomplete removal of TCS from WWTPs and the applications of TCS laden biosolids into agricultural soils, leads to TCS being distributed in aquatic and terrestrial environment. Table 5 shows the prevalence of TCS in different environmental compartments worldwide. Environmental concentrations of TCS varied with surface water type (lake/river/streams with known input of raw wastewater) ranging from 1.4–40,000 ng·L^−1^; sea water <0.001–100 ng·L^−1^; sediment (lake/river/other surface water) <100–53,000 μg·kg^−1^ dry weight (dw); sediment (marine) 0.02–35 μg·kg^−1^ dw; wastewater influent 20–86,161 ng·L^−1^; wastewater effluent 23–5370 ng·L^−1^; biosolids from WWTP 20–133,000 μg·kg^−1^ dw; activated/digested sludge 580–15,600 μg·kg^−1^ dw; pore water 0.201–328.8 μg·L^−1^ [96].

Triclosan is commonly detected in aquatic and terrestrial environments [14,98,99]. TCS is generally dumped through consumer products [100] and finally finds its way into the WWTPs. The occurrence of TCS along with other organic contaminants has been reported in Canadian municipal sewage sludge and biosolids [17,101,102]. TCS has also been identified in drinking water in certain geographical regions [103,104]. The degradation product of TCS, methyl TCS (12 μg·L^−1^) was found in one of the 22 drinking water samples from Barcelona [105]. Although WWTPs are generally highly effective in removing TCS, a small percentage of the antimicrobial is usually discharged with effluent into receiving waters. Thus, the two main sources of TCS release into the environment are: (1) discharge of WWTP effluent into receiving waters; and (2) land application of biosolids containing residues of the antimicrobial.

The efficiency of WWTP for TCS removal has been observed with an average median removal efficiency of 90% [106,107]. TCS was found to be readily degraded under aerobic conditions but was observed to be resistant to degradation under anaerobic conditions [12]. The results of the field measurements from a Swiss WWTP have indicated that during the elimination process: 79% of TCS was biologically degraded, 15% was sorbed to sludge and 6% left the plant in the final effluent at a concentration of 42 ng·L^−1^ [6]. These results are in concordance with studies conducted at various WWTPs in Germany, where 4%–10% of TCS remained dissolved in effluent [10]. Mostly, WWTP influent concentrations of the TCS range from 1.86 to 26.8 μg·L^−1^ with effluent concentrations ranging from 0.027 to 2.7 μg·L^−1^ [14,108,109]. Despite the high removal rates reported for TCS, Yang *et al.* [110] studies identified the formation of toxic byproducts during oxidation of TCS.

**Table 5:** TCS sourcing in some of the prominent environmental compartments worldwide.

Source	Sampling Source	Country	Concentration of TCS	Reference
**Surface waters**	Natural streams/rivers	USA	Up to 2.3 μg·L^−1^	[88,108]
		Switzerland	0.074 μg·L^−1^	[111]
		Germany	0.01 μg·L^−1^	[4]
		Australia	0.075 μg·L^−1^	[112]
		Japan China	0.0006–0.0059 μg·L^−1^ 0.011–0.478 μg·L^−1^	[144] [113]
	Streams with inputs of raw wastewater	Switzerland	0.011–0.098 μg·L^−1^	[6]
		USA	1.6 μg·L^−1^	[5]
	Estuarine waters	USA	0.0075 μg·L^−1^	[143]
**Sediment**	Fresh water	Switzerland	53 μg·kg^−1^	[6]
		Spain	35.7 μg·kg^−1^	[122]
	Estuarine	USA	800 μg·kg^−1^	[117]
	Marine River water	Spain China	0.27–130.7 μg·kg^−1^ 50–1330 μg·kg^−1^	[145] [114]

During 1999 to 2000, US Geological Survey detected TCS in 57.6% of streams and rivers sampled, at concentrations ranging from below the detection limit up to 2.3 μg·L^−1^ [88]. In addition, due to the partial removal efficiency of WWTPs in effluent, TCS exhibits a tendency to accumulate and persist in biosolids. According to an assessment, up to 50% of TCS in WWTP influent will remain in biosolids in WWTPs even after activated sludge treatment in combination with anaerobic biosolids digestion [14,107,114]. The concentrations of TCS in aquatic environment is governed by various factors, such as the TCS load in effluent, physical and chemical properties of TCS, characteristics of the aquatic ecosystem (pH, sediment density and organic matter content, water flow and velocity, depth), and even season and intensity of sunlight [99,100,115]. Despite the recent ban on addition of triclosan in daily use products, lower efficiency of WWTP to degrade it results in its accumulation in biosolids and hence release into the environment. The highest concentration detected 40,000 ng·L^−1^ are still half of the lowest-observed-effect concentration reported for some fishes. However, taking into account that triclosan is in use since only few years and its derivatives are much toxic and very persistent, the regulations reducing its use seem to be the most accurate decision to prevent environmental consequences.

## 7. Degradation of TCS

Table 6 provides the concentrations of TCS detected in different organisms. This antimicrobial compound has demonstrated a tendency for bioaccumulation in aquatic species [116] and it can persist in aquatic ecosystems for extended periods of time. TCS prevalence in environment mandates monitoring in surface water. Triclosan has been detected in 30-year-old sediment from Greifensee Lake in Switzerland [6]. This study provided evidence of the persistence of TCS in sediment and unravels the pattern of TCS usage.

**Table 6:** Detected concentrations of TCS in different organisms.

Organisms	Species/Sample Type	Sampling Site	TCS (μg·kg^−1^)	Reference
Algae	Filamentous algae (*Cladophora* spp.)/Whole organism	Receiving stream for the city of Denton (TX, USA) WWTP	(1) 100–150 (2) 50–400	[146] [144]
Invertebrates	Freshwater snails (*Helisoma trivolvis*)/Muscle	Receiving stream for the city of Denton (TX, USA) WWTP	50–300	[144]
Vertebrates	Rainbow trout (*Oncorhynchus mykiss*)/Bile	(1) Upstream from WWTP, Sweden (caged); (2) downstream 2 km from WWTP (caged)	(1) 710 (2) 17,000	[20]
	Breams, male (*Abramis brama*) (1) Bile (2) Muscle	(1) River sites (Netherlands) (2) River sites (Germany)	(1) 14,000–80,000 (2) 0.25–3.4	[147] [148]
	Pelagic fish/Plasma	Detroit River (USA)	0.75–10	[149]
	Atlantic bottlenose dolphins ( *Tursiops truncates*)/Plasma	(1) Estuary, South Carolina (2) Estuary, Florida	(1) 0.12–0.27 (2) 0.025–0.11	[150]
	Killer whale (Orcinus orca)/Plasma	Vancouver Aquarium Marine Science Centre	9.0	[150]

Triclosan concentrations in sediment increased between the early 1960s after its introduction until the mid-1970s, reflecting steadily increased patterns of its use. Later, an opposite trend was observed from the mid-1970s until the early 1980s, during which period, an additional secondary treatment step was introduced into most WWTPs. However, due to the popularity and increased use of TCS, again from the early 1980s, increase in TCS concentrations was observed until the present time [6]. Nevertheless, the quite high amount of TCS present in the 30-year old sediment layer from 1970 to 1971 showed that TCS degradation was very slow in the sediment. [117] also reported similar time line profile for TCS spanning last 40 years for estuarine sediments in the USA. The environmental persistence of TCS in sediments indicates the fact that antimicrobial compounds can partition into the sediments and resist degradation processes under anaerobic conditions. Moreover, sediments are the final sink of the aquatic environment and the retention of TCS in this matrix would be precarious as there are eventual possibilities of being released back into the aquatic environment by bioturbation caused by organisms or through human dredging [118,119].Although TCS possesses high chemical stability and it is extremely resistant to high and low pH, it is found to be readily degraded in the environment through photodegradation. In laboratory samples, researchers have identified eight sub-products of this photochemical process [120,121,122,123,124,125,126]. Under laboratory conditions, Latch *et al.* [21] observed TCS photoconversion to 2,8-DCDD with a yield of up to 12% at pH > 8 using different irradiation wavelengths. Authors compared the formation of 2,8-DCDD yield under laboratory conditions (purified water) with the river water spiked with the TCS. Comparable results between laboratory and realistic conditions confirmed that TCS was able to convert into 2,8-DCDD in sunlight-irradiated water sources.

**Figure 3:**
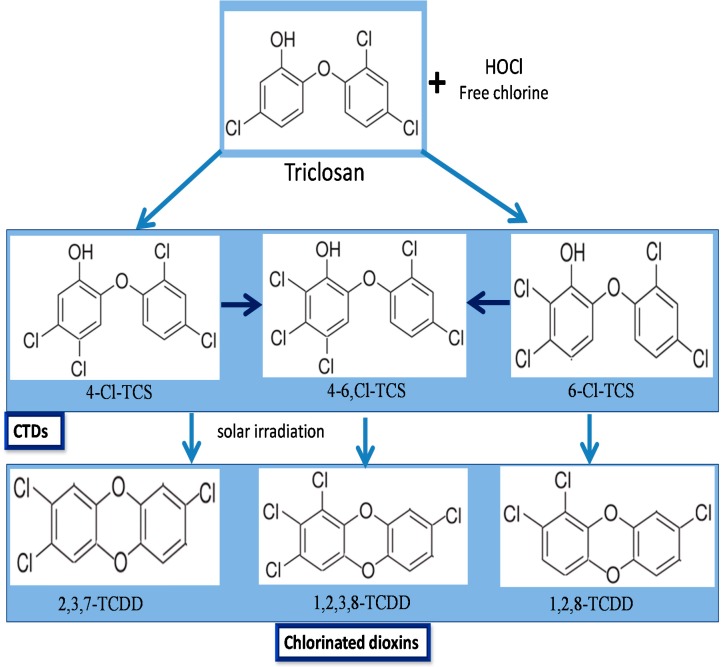
Photolytic degradation of chlorinated TCS derivatives (CTDs) to chlorinated dioxins.

Triclosan that persists in the secondary effluent after activated sludge treatment may be chemically transformed after discharge. In this sense, a disinfecting oxidant, sodium hypochlorite, a source of free chlorine is generally used in US for many purposes and could enter in contact with TCS. It is known to chlorinate the TCS phenol carbons in ortho, or para-positions generating three chlorinated TCS derivative (CTD) intermediate products: [78,122,127]. The light mediated degradation of CTDs to chlorinated dioxins is depicted in Figure 3. However, dioxin derivatives of TCS are not of public health concern mainly due to the low efficacy of direct photolysis [123]. Similarly, chloramination of TCS also forms the similar CTDs, although at a much lower rate than the free process [128]. Chlorinated derivatives of TCS, 4-Cl-TCS, 6-Cl-TCS, and 4,6-Cl-TCS were reported to be present in wastewater effluent [13,129]. Due to dispersal of TCS containing effluents in the streams, the CTDs have been also detected in the top levels of aquatic trophic chains [130], and as biomethylated analogues in fresh water samples downstream from a wastewater effluent as well as in carps living in it [131]. These results demonstrated that either CTDs are generated from TCS during wastewater disinfection with free chlorine or bypassing the standard treatments. CTDs are considered as an important environmental issue as they could may maintain or even increase the antimicrobial and endocrine-disrupting features, of TCS. Moreover, the CTDs, such as 4-Cl-TCS, 6-Cl-TCS, and 4,6-Cl-TCS are extensively reported to liberate dioxins under natural conditions of photolysis in water [87,132].

**Figure 4:**
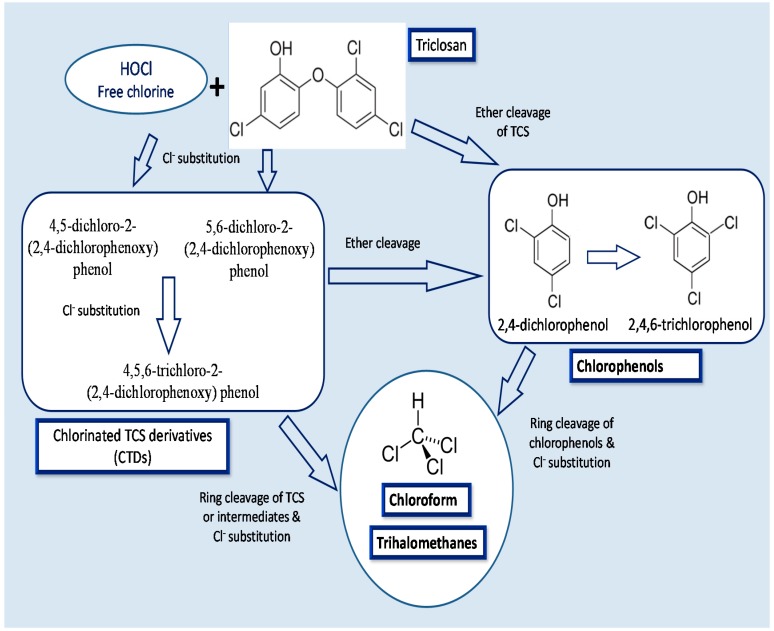
Mechanisms of TCS conversion to its intermediate products: chlorinated TCS derivatives, chlorophenols and chloroform and trihalomethanes.

The historical pattern of dioxin photoproducts of TCS and its chlorinated derivatives in sediment cores from the Mississippi river was reported by Buth *et al.* [132]. Another possible source of TCS derived dioxins comes from the solar irradiation of CTDs, leading to the formation of chlorinated dioxins. 2,8-DCDD and 2,4-dichlorophenol (2,4-DCP) are produced after photochemical degradation of TCS, when chemical by-products are exposed to UV radiation after the reaction of TCS with chlorinated H_2_O [78]. 2,4-DCP is further chlorinated to produce 2,4,6-trichlorophenol [133]. The chlorophenol intermediates are subsequently transformed to chloroform and trihalomethanes [134]. The mechanisms of CTDs transformation to chlorophenols and further to chloroform and trihalomethanes is given in Figure 4.

The repeated exposure to chlorine in water treatment facilities can chlorinate TCS. Chlorinated TCS is discharged from a WWTP, and sunlight can convert it into more toxic dioxins [135]. According to U.S.EPA, 2,4-DCP is a priority pollutant, and is considered to be toxic to fish and other aquatic organisms [56,136]. 2,4-DCP is used in the manufacture of certain pesticides, antiseptics, and disinfectants. Moreover, in the presence of solar radiation, the 2,4-DCP further breaks down and may produce more highly chlorinated dioxins [137].

Meanwhile, a study by Latch *et al.* [123] concluded that dioxin compounds formed from TCS are not of public health concern due to the low concentrations of reactive oxygen species (ROS) in natural waters and low efficacy of direct photolysis of TCS. Several bacteria such as, *Pseudomonas*, *Burkholderia* or *Sphingomonas* can degrade them in natural environments to carbon dioxide and chlorine [138,139]. Son *et al.* [140] reported that degradation of TCS through titanium dioxide photocatalysis is mediated by radicals that enhance the degradation of intermediary dioxins. Moreover, the oxidative process is maximized by hydrogen peroxide [141]. When TCS remains isolated from biotic interaction and is maintained between pH 4–9, it is stable even at 50 °C. In an aqueous solution at 25 °C and pH 7, TCS undergoes faster degradation mediated by light, reaching 50% in around 41 min. During this reaction, mainly 2,4-dichlorophenol (2,4-DCP) is produced within 4 h after treatment.

TCS is readily susceptible to degradation through photolysis in aqueous media with half-life that ranges from <1 h in abiotic conditions, to around 10 days in fresh water bodies. Moreover, its aerial half-life has been estimated to be 8 h based on reaction of TCS with photo-chemically produced hydroxyl radicals. Even though the detected concentrations as of now may not be toxic, but continuous accumulation of TCS and its by-products in the environment could reach the threshold limit which can affect all levels of the animals in the food chain.

## 8. Conclusions and Future Prospects

The ubiquitous use of triclosan and its consequent entry into the environment is of concern due to the effects it could produce if no regulations prevent its accumulation during the next decades. It and its derivatives are already present in measurable quantities, which may potentially affect water quality, impact on ecosystem and human health. Contamination of TCS has been detected in different environmental matrices including terrestrial, aquatic and biosolids resulting from WWTPs. TCS has also been found in drinking waters. There are concerns that the widespread use of TCS in various applications might lead to a preferential selection for microbial resistance to antibiotics. Microbial resistance has become an increasingly serious problem worldwide, and the continued use of biocides including TCS may exacerbate this problem. Increasing accumulation of TCS in the environment was also found to have adverse impacts on the growth of aquatic organisms. Taking into consideration the environmental and health concerns of TCS, more efforts need to be carried out for the understanding of their distribution and fate in various environmental compartments, in particular, wastewater treatment plants and sediments which are the final sinks.

## Abbreviations List

AMA: American Medical Association
APHA: American Public Health Association
CDC: Centers for Disease Control and Prevention
CTDs: Chlorinated derivatives
2,8-DCDD: 2,8-dichlorodibenzo-*p*-dioxin
2,4-DCP: 2,4-dichlorophenol
2,4-TCP: 2,4,6-trichlorophenol
ECs: Emerging contaminants
EDCs: Endocrine disrupting compounds
EPA: Environmental Protection Agency
ENR: Enoyl-acyl carrier protein reductase enzyme
EU: European Union
FDA: Food and Drug Administration
MIC: Minimum inhibitory concentrations
MRSA: Methicillin-resistant *Staphylococcus aureus*
NHANES: National Health and Nutrition Examination Survey
PACD: Photo allergic contact dermatitis
PCBs: Polychlorinated biphenyls
PCDDs: polychlorinated dibenzo-p-dioxins (dioxins)
PCDFs: polychlorinated-dibenzofurans (dibenzofurans)
PCPs: Personal care products
PhACs: Pharmaceutically active compounds
POPs: Persistent organic pollutants
ROS: Reactive oxygen species
TCDD: 2,3,7,8-tetrachlorodibenzo-*p*-dioxin
TCS: Triclosan
USEPA: United States environmental protection agency
WWTPs: Wastewater treatment plants
xxxxx: xxxxxxx
